# Evaluation of incisor position and alveolar bone relationship in untreated skeletal Class II malocclusion with different growth patterns

**DOI:** 10.6026/973206300211266

**Published:** 2025-05-31

**Authors:** Sunilkumar Nagmode, Vaishnavi Ingle, Hrushikesh Aphale, Vivek Shinde, Vinnayakk Kalaskar, Rahul Kolte

**Affiliations:** 1 Department of orthodontics and orthopedics, SMBT Dental College, Sangamner, Maharashtra, India

**Keywords:** Positions of central incisors, root inclination, alveolar bone thickness

## Abstract

The position and inclination of central incisors significantly affect treatment planning for anterior- posterior tooth movements. Hence, we analyzed 90 lateral cephalograms of untreated Class II malocclusion cases with different growth patterns. Data shows that maxillary incisors tend to occupy the anterior one-third of the alveolus in horizontal growth pattern, while mandibular incisor roots are more inclined and positioned lingually in those with a vertical growth pattern.

## Background:

The alveolar process, which maintains the teeth with the help of the roots, undergoes subsequent biologic events in bone remodeling (resorption and apposition) that enable orthodontic movement. As resorption of bone occurs in the direction of tooth movement, orthodontic therapy may be hampered by the decreased amount of alveolar bone, which can occasionally be thin or nonexistent [1, 2]. So it is crucial to determine both the alveolar bone morphology and the placement of central incisors before retraction in both the jawa. The inclination of central incisor and its relationship to the surrounding alveolar bone are easily measured with help of Lateral cephalogram. It often influences treatment options for anterior- posterior (AP) incisor motions inside the jaws [3, 4- 5]. These include movement such as retroclination, retrusion, protrusion and proclination, Furthermore; cephalometric radiographs evaluate the overall effects of the craniofacial complex's growth as well as the teeth's relative positions as a result of orthodontic movement. The alveolar morphology must be established prior to orthodontic treatment in order to prevent fenestration and/or dehiscence, which can occur above the cortical surfaces of the alveolar bone, which cause difficulty in orthodontic tooth movement [6, 7, 8- 9]. The orthodontic movement is defined by the thickness of the alveolar bone and it may have unfavorable side effects on the periodontal tissues if these limitations are challenged. The two most important orthodontic movements are dental arch expansion and buccal-lingual movements of incisor, which can decentralize teeth from the alveolar bone envelope. Depending on the morphology of alveolar bone and the extent of tooth movement, there is a higher chance of developing or aggravating alveolar defects such as bone dehiscences, fenestrations and gingival recession [10]. Induced contact between incisor roots and alveolar cortical bone can also precipitate external root resorption. Additionally, it has been demonstrated that contact between the incisive canal and maxillary incisor roots is a risk factor for external root resorption, which further restricts the range of motion that some patients may be able to achieve with their maxillay incisors [11, 12-13]. Treatment choices might be influenced by the anatomical limits for safe incisor root movement inside each jaw in order to reduce iatrogenic sequelae. Therefore, it is of interest to use lateral cephalograms to ascertain the incisor location, inclination and its relation to alveolar bone in individuals with untreated Skeletal class II malocclusion with different growth patterns.

## Materials and Methods:

Lateral Cephalogram of patients between 12 and 25 years of ages, both males and females who sought orthodontic treatment at the SMBT Dental college, Sangamner, between 2022 and 2024 having Skeletal class II malocclusion with different growth pattern were included in this Obsevative, Cross sectional type of study. The sample size was divided into 3 broad groups based on the growth pattern using the Frankfort - mandibular plane angle of the Tweeds analysis. Angle <210 - considered as Horizontal growth pattern, Angle 22° to27° - Average growth pattern, Angle 29° -Vertical growth pattern and each of these groups had 30 patients each. This group consisted of GROUP A- Untreated Skeletal class II malocclusion with Horizontal growth pattern, GROUP B- Untreated Skeletal class II malocclusion with Average growth pattern, GROUP C- Untreated Skeletal class II malocclusion with Vertical growth pattern.

## Inclusion criteria:

[1] Angle's class II molar relationship.

[2] Cephalometric image quality adequate to identify key anatomic landmarks.

## Exclusion criteria:

[1] Patients with absence of any tooth other than a third molar.

[2] 2 Patients who had undergone orthodontic treatment.

## Cephalometric tracing:

One examiner manually traced each lateral cephalogram in pencil on acetate paper. The mandible, maxilla, first molars, central incisors and the inner and outer cortical surfaces of the mandibular symphysis were among the structures. Additionally, the maxillary incisor cementoenamel junction (CEJ), maxillary incisor root midpoint (the midpoint between the CEJ and the apex along the tooth's long axis), incisal edges, occlusal planes, root apices and incisor long axes were identified and traced.

## Measurements:

[1] Maxillary central incisor root position: The distances (in millimeters) from the root midpoint to the outer cortical surface of the alveolar process on the labial (U1-lab) and palatal (U1-pal) sides will be measured perpendicular to the long axis of the tooth (Figure 1 - see PDF).

[2] Maxillary alveolar process Thickness: The total maxillary alveolar process (Mx-Alv) thickness was obtained by adding U1-lab and U1-pal distance in mm (Figure 1 - see PDF).

[3] Mandibular central incisor root position: The distances (in millimeters) from the root apex to the outer cortical surface of the alveolar process on both the labial (L1- lab) and lingual (L1-ling) sides was measured parallel to the occlusal plane (Figure 1 - see PDF)

[4] Mandibular alveolar process thickness: The total mandibular alveolar process (Md- Alv) thickness was obtained by adding L1-lab and L1-ling values in mm

[5] Incisor inclination: The acute angle formed between the long axes of the teeth and a line perpendicular to the occlusal plane gave the inclinations of the maxillary (U1- incl) and mandibular (L1-incl) central incisors (Figure 2 - see PDF)

[6] AP jaw relationship: AP jaw relationship was assessed by WITS appraisel (Figure 2 - see PDF)

One examiner completed all of the measurements. Linear measurements were taken using an electronic digital caliper, while angular measurements were taken with a manual protractor

## Statistical analysis:

Data management and analysis procedure: The data obtained was entered in Microsoft Excel sheet and further proper statistical analysis will be done.

## Data analysis plan and methods:

Statistical analysis was performed using Statistical Product and service solution (SPSS) version 16 for Windows (SPSS Inc., Chicago, IL). Descriptive quantitative data was expressed in mean and standard deviation respectively. Data normality will be checked by Shapiro-Wilk test. Intergroup comparison of means between the three groups was done with the help of one way ANOVA test and Tukey's post hoc test. Confidence interval was set at 95% and probability of alpha error set at 5%. Power of the study set at 80%.

## Results:

Total mandibular alveolar thickness (Md-Alv) (fig 1)was highest in horizontal and least in the vertical growth pattern group, however there was a little but statistically significant variation among the total maxillary alveolar thickness (Mx-Alv). The samples' AP jaw relationship (Wits) differed substantially, with the mandible being more retrognathic than the maxilla. The Horizontal group; showed significant difference between U1-pal and U1-lab thickness. U1-pal (4.63) was roughly double in magnitude to U1-lab (mean 2.83). The Horizontal group; showed significant difference between L1-lab and L1-ling thickness. L1-lab (4.63) was roughly double in magnitude to L1-ling (mean 2.83). The vertical group shows the significance difference between L1-lab (3.56) and L1-ling (2.3) thickness. U1 Incisor Inclination (fig 2) seen with descending order of Average group, Vertical group and horizontal group. L1 Incisor showed positive inclination with descending order of Vertical group, horizontal group and Average group. Difference of AP Jaw relationship - Wits appraisal seen with ascending order of Average group, horizontal group, Vertical group

Graph 1: Inter group comparison of maxillary central incisor root position and alveolar process thickness.

Graph 2: Inter group comparison of mandibular central incisor and alveolar process thickness

Graph 3: Inter group comparison difference of long-axis inclination of upper and lower Incisor.

Graph 4: Inter group comparison AP Jaw relationship Wits appraisal

## Discussion:

Much has been discussed about the crown inclination which refers to the labiolingual or buccolingual inclination of the long axis of the crown (not to the inclination of the long axis of the entire tooth [15] but root positions had not been previously evaluated. This study provides opportunity to study about Incisor position and its root inclination in subjects with untreated Class II malocclusion. This study shows that, maxillary central incisor roots had a propensity to occupy the anterior third of the alveolar process in the horizontal growers, they were found to be about centered in the alveolus among the average growers whereas in vertical growers they were placed in posterior third of the alveolar process. Highest Maxillary and mandibular alveolar bone thickness was seen with descending order of horizontal, average and vertical group in untreated skeletal class II malocclusion subjects. Numerous studies have shown that bone remodeling in response to tooth movement lags and that overall alveolar bone thickness at the apical level and labial bone thickness at the crestal level both considerably increase when the upper incisors retreat [16, 17, 18, 19-20]. This knowledge aids in the treatment planning of incisor retraction based on the alveolar bone thickness. In order to achieve the specific treatment aim, considerable incisor movements are required. Numerous aspects, mostly of a biomechanical and biologic character, should be taken into account in this situation. Bone topography and structure, tooth location inside the bone, soft tissue conditions and other factors are considered from a biologic perspective. The possible adverse consequences of orthodontic therapy, including external root resorption, bone dehiscence and fenestration and gingival retraction, are determined by the closely connected biologic and biomechanical aspects [21, 22-23].

The position and movement of lower incisors play key roles in orthodontic diagnosis and therapy. In this study we found that the root apices of lower incisors in subjects with untreated skeletal class II malocclusion with relative mandibular retrognathism, were lingually inclined and ligually placed with more alveolar bone thickness on labial side. Yamada *et al.*'s analysis of the position of mandibular incisor roots in adults with mandibular prognathism revealed that the roots' apices were closer to the labial cortical bone than to the lingual [24]. According to this study the maximum lower incisor inclination is seen in patients with the vertical growth pattern followed by the horizontal growers, depicting careful mandibular incisor retraction. Bimstein *et al.* concluded that orthodontic treatment may cause increase in thickness of buccal alveolar bone, which involves lingual positioning of the procumbent mandibular permanent central incisors [25]. Although these naturally occurring ideal root positions can be modified, if necessary, to compensate for uncorrected AP jaw position discrepancies or to improve facial profile esthetics. Majority of Class II malocclusions are due to mandibular deficiency, resulting in a retrusion of the chin. According to a study done by Manni *et al.*, Herbst treatment proved beneficial by controlling the position of maxillary and mandibular incisors. It improved mandibular length and facial esthetics in growing patients with skeletal Class II malocclusion [26]. Angle's class 2 molar relations in centric relation and the wits assessment were employed to distinguish the Class II skeletal malocclusion samples; nevertheless, none of these methods may have been the best way to gauge AP jaw disparity in every situation. Excess overjet was evident in several of the skeletal class II sample members, suggesting that there were little natural dental compensation; results could differ in a population with a wider range of malocclusions.

## Conclusion:

Highest maxillary and mandibular alveolar bone thickness is seen with descending order of horizontal group, average group and vertical group. In untreated individuals with class II malocclusion, maxillary central incisor roots in the alveolar process occupy the anterior one-third in horizontal and posterior one third in vertical growers. The root apices are roughly centered in the alveolus among patients with average growth pattern. In untreated individuals with Class II malocclusion and relative mandibular retrognathism, the mandibular central incisors tend to be more forwardly inclined and positioned closer to the tongue. Additionally, in vertical growers, the root apices are located further posteriorly compared to those in horizontal and average growth patterns.
